# Persistent Atrial Septum Defect Despite Placement of Two Amplatzer Septal Occluders

**DOI:** 10.15171/jcvtr.2015.37

**Published:** 2015-11-28

**Authors:** Kay B. Leissner, Jahan Porhomayon, Nader D. Nader

**Affiliations:** ^1^ Department of Anesthesiology, Harvard Medical School, Boston, MA, USA; ^2^ Department of Anesthesiology, Boston University School of Medicine, Boston, MA, USA; ^3^ Department of Anesthesiology and Critical Care Medicine, University at Buffalo, Buffalo, NY, USA

**Keywords:** Atrial Septal Defect, Amplatzer, Percutaneous Occluders

## Abstract

Herein, we are presenting a case of persistent interatrial septal defect diagnosed during coronary artery bypass grafting (CABG). Twice, attempts had been made to close this shunt using amplatzer septal occlude. However, percutaneous technique had failed in both occasions. The patient presented with chest pain 4 years after the second attempt and required urgent CABG. Persistent shunt was repaired during surgery.

## Case Presentation

A 55-year-old female presented to the hospital with chest pain. Her medical history included delayed closure of a secundum atrial septum defect (ASD) with an Amplatzer Septal Occluder (ASO) at age 50 and a repeated closure of a persistent ASD with a second ASO one year later. Cardiac catheterization revealed 2-vessel disease requiring urgent coronary artery bypass grafting (CABG).

**Figure 1:**
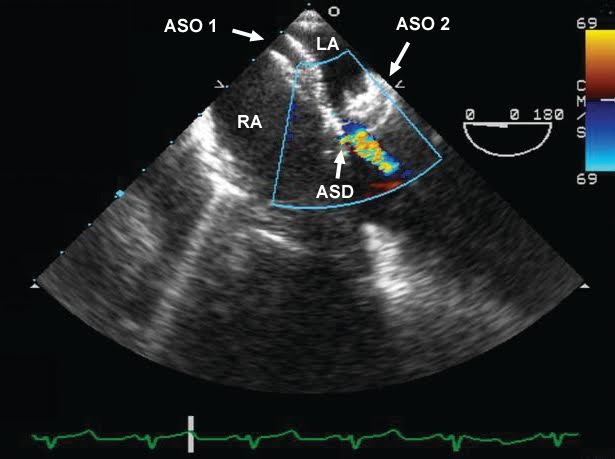
Modified 4-chamber view showing two ASD closure devices and the remaining shunt in interatrial septum.

**Figure 2:**
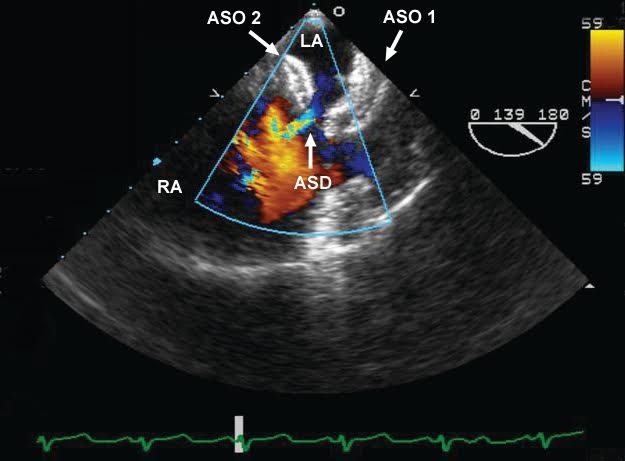
Modified bicaval view showing two ASD closure devices and the remaining shunt in interatrial septum.

Intraoperative transesophageal echocardiography (TEE) revealed mild left ventricular (LV) hypertrophy with an estimated ejection fraction (EF) of 55% and mild posterior hypokinesia. The right atrium (RA) and right ventricle (RV) were moderately dilated and RV function was normal. The interatrial septum was visualized in the midesopageal (ME) 4-chamber view, the ME bicaval view and a modified ME bicaval view at 135°. The two previously placed ASO devices were clearly visualized. Color Doppler imaging demonstrated left to right flow between the two ASO devices indicative of a residual ASD (Figures 1 and 2). The surgeon was informed and it was decided to repair the ASD. Superior and inferior vena cava cannulae were inserted and the two ASO devices were excised. A pericardial patch was then sutured to the remaining interatrial septum and a two vessel CABG was performed using left internal mammary artery graft for left anterior descending artery and also saphenous vein graft for obtuse marginal artery. The patient had an uncomplicated postoperative course and subsequent transthoracic echocardiography showed no residual ASD.

## Discussion

ASD is the second most common congenital lesion in adults after bicuspid aortic valve. There are four types of ASD based on the location: primum, secundum, coronary sinus and the sinus venosus ASD.^1^ Defects in the area of the foramen ovalis are secundum type ASD, which result from poor growth of the secundum septum or excessive absorption of the primum septum and accounts for 75% of all ASDs.^2^

Frequently patients with significant ASD shunt flow become symptomatic at the age of 30–40 years with dyspnea, palpitations, edema, and recurrent respiratory infections. Typical complications are: atrial fibrillation, heart failure, and thromboembolic events.^3,4^ There are three main indications for closure of an ASD: the development of clinical symptoms, a high rate of shunt flow, and thromboembolism. A pulmonary to systemic blood flow ratio (Qp/Qs) >2:1 is an established indication for correction, though some authors have advocated lower thresholds.^5,6^ ASD may be closed surgically or percutaneously.

Nonoperative device closure is applicable only to secundum ASD. More than one device may be inserted to close multiple ASD.^7-9^ Prior to the procedure, TEE is used to document the type and sizeof the ASD, the shunt flow direction and ratio, and the presence of anomalous pulmonary venous return. Thefunctional importance of the ASD can be estimated by thesize of the RA and RV, interventricular septal flattening in diastole (RV volume overload)or systole (RV pressure overload), paradoxical septal motion (RV volume overload),and an estimation of the shuntratio.^7^

While former devices (ASDOS, SIDERIS, and CardioSEAL) had a major complication rate of 17.6%, the new devices (ASO, Helex, STARflex, and Premere) demonstrated their safety with an overall complication rate of 0.8%.^2^ The ASO consists of two round disks made of Nitinol wire mesh that are linked together by a short connecting waist.^10^ It comes in various sizes from 4 mm to 38 mm. The ASO has several advantages over other devices: It can be delivered through smaller catheters, it is self-centering but can be repositioned easily, round retention disks extend radially beyond the defect resulting in a smaller overall size and firmer contact with the atrial septum, thereby enhancing endothelialization, and reducing the risk of residual shunting.^10^

TEEand fluoroscopy are used to guide ASO placement. For successful ASO closure, the ASD is ideally less than 30 mm in diameter with a rim of tissue around the defect of at least 5 mm to prevent obstruction of the coronary sinus, pulmonary veins, vena cava, or atrioventricular valves.^11,12^ For correct ASO sizing the diameters of defects are measured in various imaging planes including theme four-chamber view (0°), the aortic short axis view (45°), and the bicaval view (90°). The maximal diameter of the defect is measured during end-diastole in at least two perpendicular views. Real-time three-dimensional TEE has been successfully used in guiding the ASO implantation procedure.^13^

ASD occlusion rate at three months was 99% in patients treated with the ASO have been reported.^14,15^ The total ASD occlusion rate at three months was 99%. In one recent study multiple ASD device implants were safely employed with excellent outcomes.^2^

In this case the patient had 2 previous ASO implanted, but a significant left to right shunt was visualized by TEE during color Doppler interrogation of the interatrial septum. The operation was changed to include the excision of the two ASO and closing the ASD with pericardial tissue. This case emphasizes the need of perioperative echocardiography in patients with previous non-surgical ASD device closure.

## Ethical Issues

The study was approval by local Ethics Committee.

## Competing Interests

No potential conflicts of interest including commercial relationships exist.
